# Impulsivity in Parkinson’s Disease Is Associated With Alterations in Affective and Sensorimotor Striatal Networks

**DOI:** 10.3389/fneur.2018.00279

**Published:** 2018-04-26

**Authors:** Marit F. L. Ruitenberg, Tina Wu, Bruno B. Averbeck, Kelvin L. Chou, Vincent Koppelmans, Rachael D. Seidler

**Affiliations:** ^1^School of Kinesiology, University of Michigan, Ann Arbor, MI, United States; ^2^National Institute of Mental Health, Bethesda, MD, United States; ^3^Department of Neurology, University of Michigan Health System, Ann Arbor, MI, United States; ^4^Department of Psychology, University of Michigan, Ann Arbor, MI, United States

**Keywords:** Parkinson’s disease, impulsivity, basal ganglia, affective striatum, sensorimotor striatum

## Abstract

A subset of patients with Parkinson’s disease (PD) experiences problems with impulse control, characterized by a loss of voluntary control over impulses, drives, or temptations regarding excessive hedonic behavior. The present study aimed to better understand the neural basis of such impulse control disorders (ICDs) in PD. We collected resting-state functional connectivity and structural MRI data from 21 PD patients with ICDs and 30 patients without such disorders. To assess impulsivity, all patients completed the Barratt Impulsiveness Scale and performed an information-gathering task. MRI results demonstrated substantial differences in neural characteristics between PD patients with and without ICDs. Results showed that impulsivity was linked to alterations in affective basal ganglia circuitries. Specifically, reduced frontal–striatal connectivity and GPe volume were associated with more impulsivity. We suggest that these changes affect decision making and result in a preference for risky or inappropriate actions. Results further showed that impulsivity was linked to alterations in sensorimotor striatal networks. Enhanced connectivity within this network and larger putamen volume were associated with more impulsivity. We propose that these changes affect sensorimotor processing such that patients have a greater propensity to act. Our findings suggest that the two mechanisms jointly contribute to impulsive behaviors in PD.

## Introduction

Approximately 6–15.5% of Parkinson’s disease (PD) patients experience problems with impulse control (1–3). Impulse control disorders (ICDs) are characterized by a loss of voluntary control over impulses, drives, or temptations to engage in excessive hedonic behavior that interferes with daily functioning and is harmful to the patient and/or others. The most common ICDs in PD are pathological gambling, hypersexual behavior, compulsive buying, and compulsive eating [e.g., Ref. (2, 3)]. There are indications that dopamine agonists are linked to ICDs in PD [e.g., Ref. (2, 4–6)], although not all studies support this claim (7). Understanding the neural bases of ICDs in PD could provide biomarkers for tracking ICD risk and recovery.

PD patients with ICDs compared to those without have a reduced reward circuitry functional connectivity between the striatum and anterior cingulate cortex (ACC) (8, 9). Atypical functioning within this circuitry has also been associated with addictive behaviors and impaired inhibitory control [for a review, see Ref. (10)]. While some studies found no structural gray matter (GM) differences between PD patients with and without ICDs (11, 12), another observed a reduced GM volume in orbitofrontal cortex (OFC) of PD patients with pathological gambling compared to those without (13). Also, compared to healthy participants, PD patients with pathological gambling showed a smaller GM volume in the OFC and ACC, among other structures. The OFC is also part of the reward circuitry and involved in detecting, encoding, and updating the reward value of events, thereby influencing future decision making (14). Interestingly, the studies reporting no GM differences did find cortical thickness increases in the ACC and OFC in PD patients with ICDs compared to those without (11, 12), which were positively correlated with ICD severity (12). These discrepancies between GM volume and cortical thickness measures are difficult to interpret [cf Ref. (15)]. This difference aside, the literature indicates that ICDs in PD are associated with neurostructural and functional reward circuitry changes.

At the behavioral level, PD patients with ICDs gather less information before making decisions than patients without ICDs (16). Previous studies demonstrated that healthy participants who gathered more information before decision making engaged a parietal–frontal network more strongly (17) and had larger GM volumes in areas within this network (18) than participants who gathered less information. The overlap between anomalies in functional connectivity and structural brain properties in PD patients with ICDs on the one hand, and networks and structures involved in information gathering and decision making in healthy participants on the other hand suggests that differences in functional connectivity and structural properties between PD patients with and without ICDs may be associated with behavioral differences in impulsivity. To date, investigations of the neural correlates of impulsivity in PD have primarily focused on reward and decision-making circuitries, with less focus on sensorimotor striatal pathways. However, alterations in sensorimotor pathways likely contribute to impulsivity too, as cerebellar volume has been linked to impulsive tendencies in psychiatric patients (19) and abnormal premotor cortical connectivity to impulsivity in juveniles (20). Therefore, the present study aimed to investigate differences in both affective and sensorimotor striatal circuitries between PD patients with and without ICDS and their association with impulsive behaviors. We had two groups of PD patients (with vs. without ICDs) perform an information-gathering task in which they chose between evidence-seeking actions and actions leading to potential rewards or losses (16, 17). Using such a well-characterized task to study the neural bases of ICDs in PD is a novel approach, as previous studies have compared only brain indices of PD patients with and without ICDs and did not include behavioral assessments (other than scores on impulsivity questionnaires). We collected resting-state functional connectivity MRI (rs-fcMRI) and structural MRI data to (1) investigate structural and resting-state functional connectivity differences between PD patients with and without ICDs, and (II) evaluate whether individual differences in these neural measures were associated with behavioral impulsivity (i.e., information-gathering task performance and impulsiveness questionnaire score; see Materials and Methods). In line with previous studies, we expected PD patients with ICD to exhibit a reduced corticostriatal connectivity (especially between striatum and ACC). Moreover, we hypothesized that less information gathering and higher impulsivity scores would be associated with reduced network connectivity strength and smaller GM volume in affective striatum but increases in sensorimotor striatum.

## Materials and Methods

### Participants

Fifty-one mild to moderate-stage PD patients [aged 40–74 years, Hoehn and Yahr stages 1–3 (21)] participated in the study. Using the Questionnaire for Impulsive–Compulsive Disorders in PD [QUIP (22)], we classified 21 patients as having ICD (ICD+ group) and 30 as not (ICD− group). The ICD+ group indicated pathologic gambling (*n* = 1), compulsive sexual behaviors (*n* = 9), compulsive buying (*n* = 7), compulsive eating (*n* = 11), or other compulsive behaviors (*n* = 6; nine patients indicated a combination of two or more behaviors). All patients provided written informed consent in accordance with the Declaration of Helsinki. The study was approved by the medical institutional review board of the University of Michigan.

### Experimental Task and Procedure

Patients were tested while their symptoms were being well controlled by dopamine replacement medication. The Unified Parkinson’s Disease Rating Scale (UPDRS) motor subscale was used to assess motor symptoms. Patients completed the Barratt Impulsiveness Scale (BIS) questionnaire, for which we determined a total score as well as separate scores for the three factors: attentional impulsiveness, motor impulsiveness, and non-planning impulsiveness (23, 24). We also used the Montreal Cognitive Assessment (MoCA) (25) and the National Adult Reading Test-Revised (NART-R) (26) to assess patients’ global cognitive abilities and verbal intelligence, respectively. After completing these assessments, patients performed the “beads task,” which has been used in previous investigations on evidence-seeking and impulsivity in both healthy and clinical populations [e.g., Ref. (16–18, 27)]. Participants were instructed to imagine two urns filled with blue and green beads, with one (the “blue urn”) containing mostly blue beads and the other (the “green urn”) containing mostly green beads. They were informed that in each trial, they would view a sequence of beads drawn from one of the urns and that they had to infer from which “hidden urn” each sequence of bead colors was drawn. They were allowed to practice the task before they performed it in the scanner.

**Figure 1:**
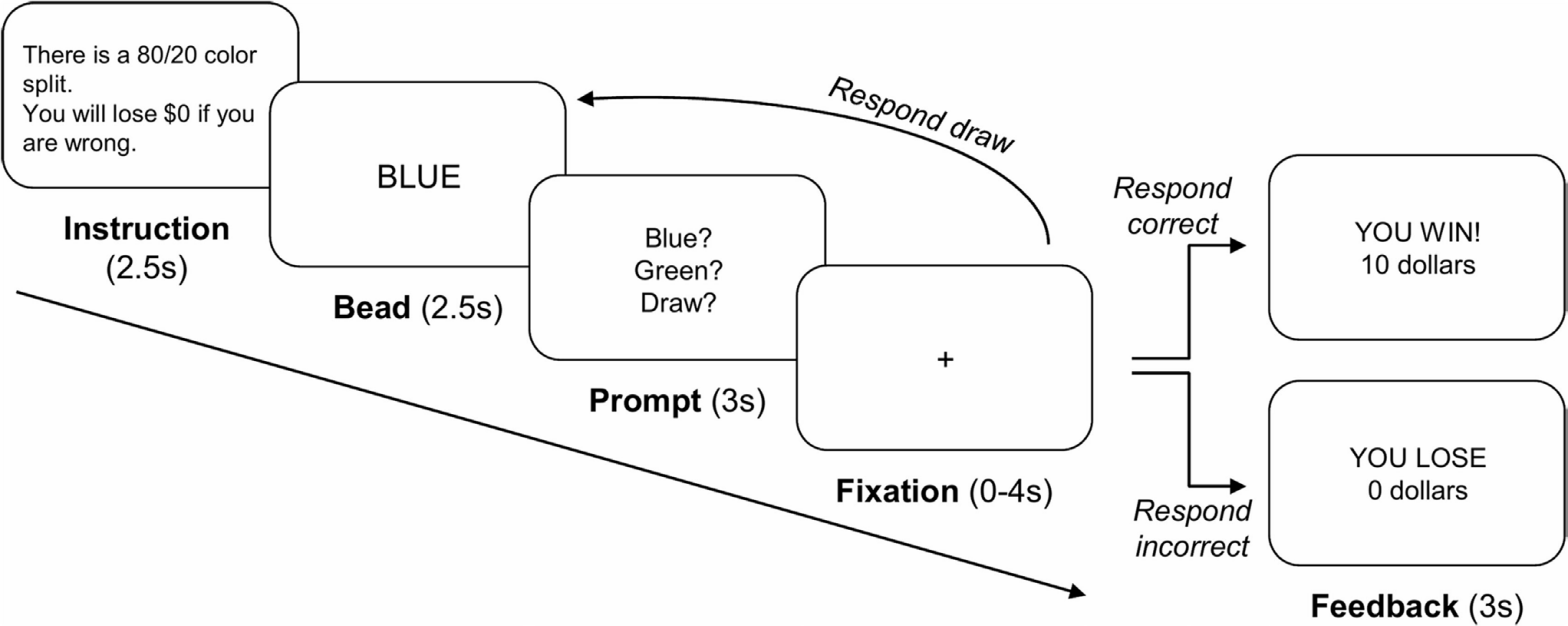
Overview of the beads task. Each sequence started with an instruction screen showing the proportion of bead colors in the two urns (80/20 or 60/40 color split) and the cost for an incorrect urn choice ($10 or $0). Participants then viewed a bead color and indicated that they either chose to draw another bead ($0.25 cost) or chose an urn. Upon a draw choice, another bead of the sequence was presented. Upon an urn choice, a feedback screen was presented, which displayed whether the participant chose the correct or incorrect urn and how much money they won or lost during that sequence.

As illustrated in Figure 1, each sequence began with an instruction screen (2.5 s) showing the proportion of bead colors in the two urns (either 80/20 or 60/40 color split) and the cost for an incorrect urn choice (either $10 or $0). Participants then viewed a bead color (2.5 s) followed by a response prompt (3 s), at which point they decided either to choose an urn or to draw another bead (maximum of nine draw choices per sequence). To reduce working memory demands, we displayed the previously drawn beads’ color as dots on the screen. After a 0- to 4-s randomly jittered fixation period, the next stimulus was either a new bead color or a feedback screen (3 s), depending on the participant’s response. The feedback screen informed participants whether they were correct or incorrect and how much money they won or lost during that sequence. Participants completed six runs of 16 bead sequences, with four sequences in each cell of a 2 × 2 factorial design with bead probability (80/20 or 60/40) and loss ($10 or $0) as repeated measure factors. Participants were informed that they would accumulate wins and losses throughout the task. They always incurred a $0.25 cost for each draw choice and won $10 for each correct urn choice. After the experiment, participants were paid 5% of the total amount that they won (*M* = $29.03, SE = $0.79).

Stimulus presentation, timing, and behavioral data registration were controlled by Cogent software (http://www.vislab.ucl.ac.uk/cogent.php) running in the Matlab environment. Patients responded *via* an MRI-compatible claw, with separate buttons designated for choosing the blue urn, the green urn, or drawing another bead. For each patient, structural and rs-fcMRI scans were obtained prior to completion of the task in the scanner; task-based fMRI results will be presented elsewhere. All patients performed the experimental task and other assessments in a single test session which lasted about 90–120 min.

### MRI Acquisition

Resting-state functional connectivity images were acquired on a 3-T GE Signa MRI scanner using a gradient-echo T2*-weighted gradient-echo pulse sequence. The field of view (FOV) was 220 mm × 220 mm with a 64 × 64 × 43 matrix resulting in an in-plane voxel resolution of 3.44 mm × 3.44 mm × 3.00 mm (for two subjects, the matrix was 64 × 64 × 35 and the voxel size was 3.44 mm × 3.44 mm × 3.50 mm.). The repeat time to accomplish a full volume (TR) was 2,000 ms, the echo time (TE) was 30 ms, and the flip angle was 90°. The slices were collected in an interleaved multi-slice mode (no slice gap), covering the whole brain (scan duration of ~8 min). Structural images were acquired using a T1-weighted spin-echo pulse sequence (TR = 540 ms, TE = 2.32 ms, flip angle = 15°) with an FOV of 220 mm × 220 mm and with a 256 × 256 × 124 matrix, resulting in an in-plane voxel resolution of 1.0156 mm × 1.0156 mm × 1.20 mm (scan duration of ~10 min).

### MRI Data Processing

We used Statistical Parametric Mapping software version 12 (SPM12; Wellcome Trust Center for Neuroimaging) running in the Matlab R2015b environment (Mathworks, Sherborn, MA, USA) for slice timing and motion correction. Slice timing correction to the first slice was performed using SPM’s sinc interpolation. Head motion correction was performed by co-registering each image to the mean EPI image. To examine outliers due to spiking and motion, and additionally to estimate Euclidian motion, we used the Artifact Detection Tool software package [ART (web.mit.edu/swg/software.htm)]. None of the patients showed head motion (translation and rotation about each of the axes) greater than 3 mm during the experiment.

Functional connectivity MRI data were normalized using Advanced Normalization Tools v2.1.0 rc3 [ANTs (28)], following a multistep approach in which we (1) preprocessed the T1-weighted image, (2) calculated the warp parameters from the T1-weighted image to an MNI152 template, and (3) applied these warp parameters to the fcMRI data. First, for preprocessing image intensity, non-uniformity correction was estimated and applied to all T1 images within a subject-specific brain mask using N4ITK (29). The brain masks were created using FSL’s Brain Extraction Tool (30) with robust brain center estimation and a fractional intensity threshold of 0.2. For each patient, we co-registered the structural preprocessed T1-weighted image to the mean functional image. Because the side of the body that was predominantly affected by the disease differed among patients, we flipped the images of subjects with left-sided motor symptom dominance along the *x*-axis (i.e., left–right direction) prior to normalization (10 patients in the ICD+ and 11 patients in the ICD− group). This ensured that in our analyses, the left hemisphere in all images reflects the patients’ most disease-affected hemisphere. Next, we spatially normalized the co-registered skull-stripped T1 images to the MNI152 template (31). The warp from the single subject T1 to the MNI152 template was calculated using ANTs with cross-correlation as the similarity metric and symmetric normalization as the transformation model (28). Finally, the resulting normalization parameters were applied to the patient’s functional images, which were then spatially smoothed with a Gaussian kernel with a sigma of 3.4 mm (i.e., 8-mm FWHM) using FMRIB Software Library (32).

### Behavioral Analyses

We used the number of draw choices and the proportion of correct urn choices as performance measures for the beads task [cf Ref. (16, 17)]. We performed a mixed ANOVA on each measure with Group (2; ICD+ vs. ICD−) as a between-subject variable and probability (2; 80/20 vs. 60/40) and loss (2; $10 vs. $0) as within-subject variables. Shapiro–Wilk tests confirmed that the data of both measures were normally distributed in each group (*p*’s > 0.11).

### Functional Connectivity Analyses

We used the CONN toolbox [version 16.a (33)] with default settings (34) to perform our rs-fcMRI analyses. Residual head motion realignment parameters and motion outliers as determined during preprocessing by the ART toolbox and signals from the white matter and cerebrospinal fluid were regressed out during the calculation of functional connectivity maps. For the first-level analysis, we used six regions of interest (ROIs) to examine differences in functional connectivity between the ICD+ and ICD− groups. We identified five basal ganglia ROIs from the Basal Ganglia Human Area Template [BGHAT (35)]: the left putamen, caudate, external and internal portions of the globus pallidus, and subthalamic nucleus. Because a previous study reported that activation in parietal cortex during making of an urn choice is associated with individual differences in the average number of draws (17), we also included a parietal ROI of 4-mm radius around the peak coordinates (40, −40, 40). The CONN toolbox determined the mean time series of each ROI. Next, the software calculated Pearson’s correlation coefficients between this mean time series of each ROI and the time series of each remaining voxel.

For the second-level analysis, performed in SPM12, a two-sample *t*-test was applied to evaluate group differences. We used a one-sample *t*-test to examine associations between behavioral performance and connectivity between the ROIs and the rest of the brain. We entered the average number of draw choices and the BIS score (total score as well as scores on each of the three factors) as predictors and included age as a covariate of no interest. All effects were evaluated using a statistical threshold of *p* < 0.0005 (uncorrected for multiple comparisons) and a minimum cluster size of 10 voxels; a few effects were significant at a family-wise error (FWE) corrected *p* < 0.05, as indicated in the tables and text. We used the Harvard-Oxford Cortical and Subcortical Structural Atlases (36) and the probabilistic cerebellar atlas (37) for anatomical localization.

### Structural Analyses

We used voxel-based morphometry (toolbox in SPM5) to evaluate group differences in structural properties and associations with behavior. Each subject’s SPGR scan was segmented into GM, WM, CSF, and other nonbrain partitions and warped to MNI space. Warped images were modulated to allow for tests of GM volumes and were then smoothed using a 10-mm FWHM Gaussian kernel. To evaluate differences in striatal GM volume between the ICD+ and ICD− groups, we performed a two-sample *t*-test within the volumes of the bilateral summed BGHAT ROIs. In addition, we examined associations between GM volume and the average number of draw choices and the BIS score; age was included as a covariate of no interest. Tests were evaluated using a cluster size threshold of 10 voxels and *p* < 0.005 (uncorrected); again, a few effects were significant at FWE-corrected *p* < 0.05 as reported in the text and tables.

## Results

Table 1 shows the group demographic and clinical characteristics. The groups did not differ significantly on age, gender, age of PD onset, disease duration, UPDRS motor subscale scores, or levodopa equivalent dose (*ps* > 0.19). The ICD+ group scored significantly higher on the BIS questionnaire than the ICD− group, *t*(49) = 2.32, *p* = 0.025. The BIS scores are consistent with those previously reported for PD patients with and without impulsivity (38), and scores of the ICD− group fall within the normal limits (24). When differentiating between the different BIS factors, we observed that the ICD groups mainly differed on motor impulsiveness and less so on attentional and non-planning impulsiveness (see Table 1). MoCA scores ranged from 24 to 30 and did not differ significantly between the two patient groups (*p* = 0.17). NART-R scores indicated that IQ estimates were within the normal range and did not differ significantly between groups (*p* = 0.89).

**Table 1:** Overview of the demographic and clinical characteristics of the impulse control disorder (ICD)+ and ICD− groups (mean ± SD).

Measure	PD ICD+	PD ICD−	Group difference
# subjects	21	30	
Age (years)	60 ± 5	62 ± 8	*t*(49) = −3.92, *p* = 0.69
Gender	7 F/14 M	11 F/19 M	χ^2^(1) = 0.06, *p* = 0.80
Handedness	3 L/18 R	4 L/26 R	χ^2^(1) = 0.01, *p* = 0.92
Age of PD onset (years)^a^	55.9 ± 6.2	58.1 ± 8.4	*t*(47) = −0.99, *p* = 0.32
Disease duration (months)^a^	57.3 ± 30.7	44.2 ± 37.7	*t*(47) = 1.31, *p* = 0.19
LED (mg)	561 ± 322	486 ± 332	*t*(49) = 0.80, *p* = 0.42
UPDRS motor	25.95 ± 9.92	25.33 ± 9.49	*t*(49) = 0.22, *p* = 0.82
BIS total score	61.90 ± 15.16	54.10 ± 8.85	*t*(49) = 2.32, *p* = 0.025
Attentional impulsiveness	16.33 ± 5.25	14.00 ± 3.47	*t*(49) = 1.91, *p* = 0.062
Motor impulsiveness	21.90 ± 4.93	19.10 ± 2.99	*t*(49) = 2.53, *p* = 0.015
Non-planning impulsiveness	23.67 ± 6.39	21.00 ± 4.55	*t*(49) = 1.74, *p* = 0.088
MoCA	27.95 ± 1.59	27.33 ± 1.54	*t*(49) = 1.39, *p* = 0.17
NART-R (FSIQ score)	112.49 ± 7.58	112.25 ± 5.56	*t*(49) = 0.13, *p* = 0.89

*^a^Data from two patients in the ICD− group were missing*.

*PD, Parkinson’s disease; BIS, Barratt Impulsiveness Scale; MoCA, Montreal Cognitive Assessment; NART-R, National Adult Reading Test-Revised; FSIQ, Full Scale IQ; LED, Levodopa Equivalent Dose; UPDRS, Unified Parkinson’s Disease Rating Scale*.

### Behavioral Results

Participants drew more beads during sequences with 60/40 compared to 80/20 probability (2.65 vs. 1.59 draws), *F*(1,49) = 99.48, *p* < 0.001, η*_p_*^2^ = 0.67. They also drew more beads during $10 loss than $0 loss trials (2.33 vs. 1.90 draws), *F*(1,49) = 29.79, *p* < 0.001, η*_p_*^2^ = 0.38. Results showed no significant effects of group (ps > 0.17). Participants more often chose the correct urn during sequences with 80/20 than with 60/40 probability (0.89 vs. 0.72), *F*(1,49) = 121.51, *p* < 0.001, η*_p_*^2^ = 0.71. Again, we observed no significant group effects (*ps* > 0.33).

### Functional Connectivity Results

**Figure 2:**
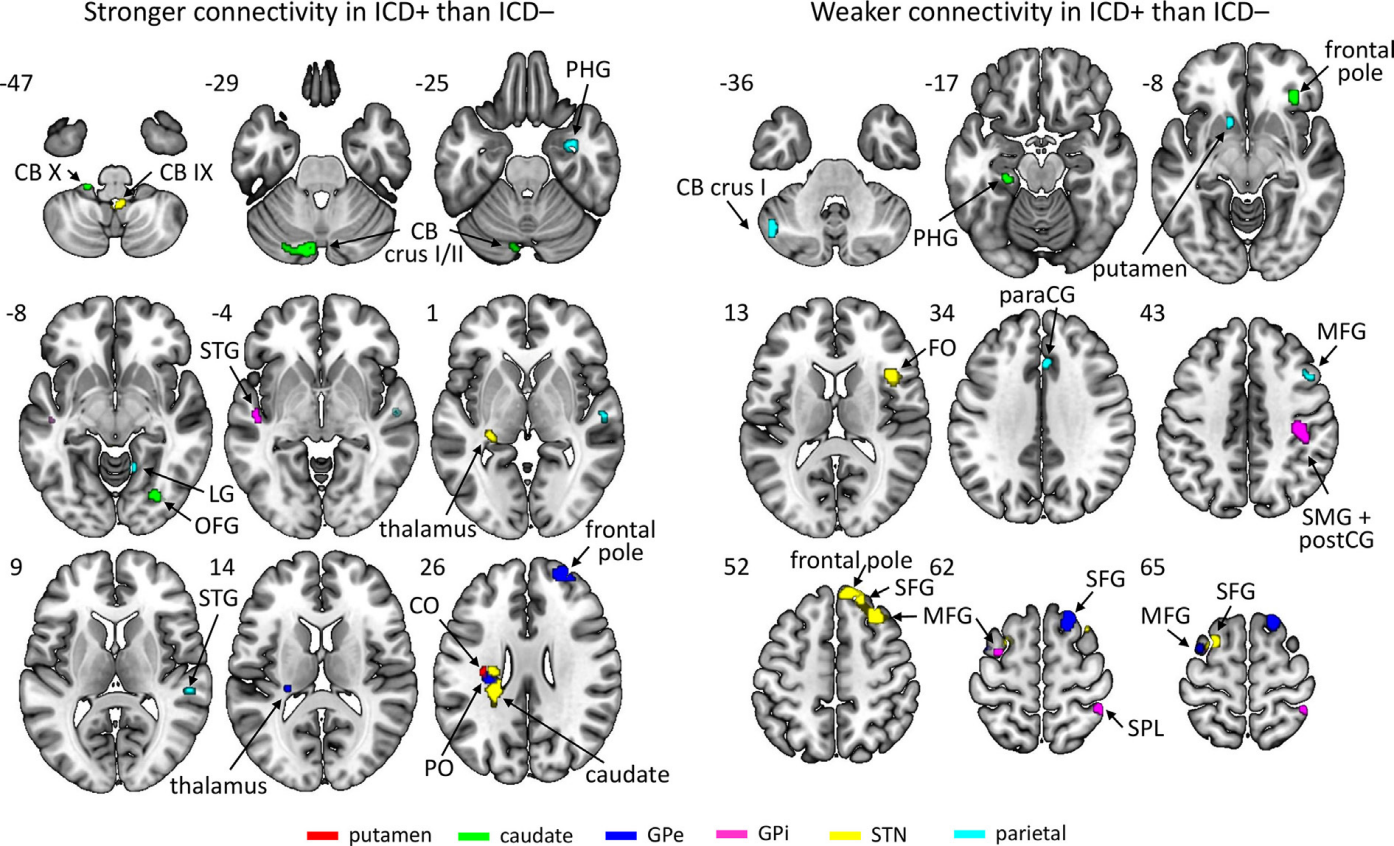
Regions showing stronger (left) or weaker (right) connectivity with their respective region of interest in the impulse control disorder (ICD)+ group than in the ICD− group. The key for the abbreviations can be found in the notes of Table 2.

Results revealed an increased connectivity between the left STN and the left parietal operculum in the ICD+ group that was significant at an FWE-corrected threshold. Furthermore, patients with higher BIS scores (i.e., more impulsivity) exhibited a weaker connectivity between the left putamen and the right inferior temporal gyrus. At the conservative uncorrected threshold, connectivity differed significantly between the ICD+ and ICD− groups for all ROIs (see Figure 2; Table 2). The left putamen showed a stronger connectivity with the central operculum in the ICD+ compared to that in the ICD− group. The left caudate showed a stronger connectivity with the occipital fusiform gyrus and various cerebellar regions in the ICD+ compared to that in the ICD− group, but a weaker connectivity with the right frontal pole, superior parietal lobule, and parahippocampal gyrus. Functional connectivity between the left GPe and various frontal cortical areas was weaker in the ICD+ group compared to ICD−. For the left GPi, the ICD+ group showed a stronger connectivity with the left superior temporal gyrus, but a weaker connectivity with various frontal and parietal areas. The ICD+ group further showed a stronger connectivity between the left STN ROI and the left caudate, and some cerebellar regions. However, the ICD+ group showed a weaker connectivity between the left STN and various frontal areas. Finally, results showed a stronger connectivity between the parietal ROI and various temporal areas in the ICD+ group, but a weaker connectivity between this ROI and the paracingulate gyrus, middle frontal gyrus, and several subcortical areas.

**Table 2:** Regions that showed differences in connectivity to the region of interest (ROI) between the impulse control disorder (ICD)+ group and the ICD− group.

ROI	ICD+ > ICD−				ICD+ < ICD−			
	Anatomic location	Coordinates of peak	Cluster size	*Z*-score	Anatomic location	Coordinates of peak	Cluster size	*Z*-score
L putamen	L CO (S1)	−34, −18, 26	10	3.43	–	–	–	–

L caudate	L CB lob X R OFG L CB crus II LCB crus I	−20, −34, −46 30, −74, −8 −10, −82, −30 −24, −82, −32	51 26 93 (93)	4.30 3.82 3.80 3.77	R frontal pole L SPL L PHG	34, 34, −8 −18, −50, 78 −26, −28, −18	49 11 15	4.03 3.62 3.54

L GPe	R frontal pole L thalamus L PO	26, 56, 26 −24, −30, 16 −30, −22, 26	98 57 (57)	4.21 3.56 3.48	R SFG R SFG L MFG	20, 22, 62 6, 40, 62 −34, 0, 68	96 11 15	4.12 3.51 3.49

L GPi	L STG	−48, −18, −4	14	3.71	R postCG R SMG R SPL L MFG	38, −24, 42 44, −32, 44 42, −42, 64 −32, −2, 62	79 (79) 29 13	3.80 3.54 3.74 3.62

L STN	L PO^a^ L caudate R CB lob IX L thalamus L CB lob I-IV	−22, −30, 22 −26, −16, 26 4, −48, −46 −28, −30, 2 −4, −42, −6	189 (189) 27 18 15	4.86 3.62 3.93 3.89 3.73	R MFG R frontal pole R SFG R FO L SFG L OFC	30, 26, 54 8, 42, 52 20, 32, 56 44, 12, 10 −24, 8, 66 −16, 14, −30	286 (286) (286) 70 47 11	4.24 3.90 3.77 4.06 4.04 3.55

R parietal	R PHG R LG R STG R STG	32, −6, −22 14, −54, −8 56, −32, 8 56, −14, −2	47 11 11 18	4.29 3.73 3.56 3.47	Brain stem L CB crus I L putamen R paraCG R MFG	−2, −40, −52 −46, −62, −34 −14, 12, −8 4, 24, 34 46, 14, 44	27 29 12 13 15	4.06 3.70 3.60 3.55 3.44

*Cluster sizes between parentheses denote additional peaks within the same cluster as listed in the row immediately preceding*.

*GPe/GPi, external/internal portion of the globus pallidus; STN, subthalamic nucleus; CO, central operculum; S1, primary sensory cortex; CB, cerebellum; OFG, occipital fusiform gyrus; SPL, superior parietal lobule; PHG, parahippocampal gyrus; PO, parietal operculum; SFG, superior frontal gyrus; MFG, middle frontal gyrus; STG, superior temporal gyrus; postCG, postcentral gyrus; SMG, supramarginal gyrus; FO, frontal operculum; OFC, orbitofrontal cortex; LG, lingual gyrus; paraCG, paracingulate gyrus*.

*^a^Remained significant at FWE-corrected p < 0.05*.

**Figure 3:**
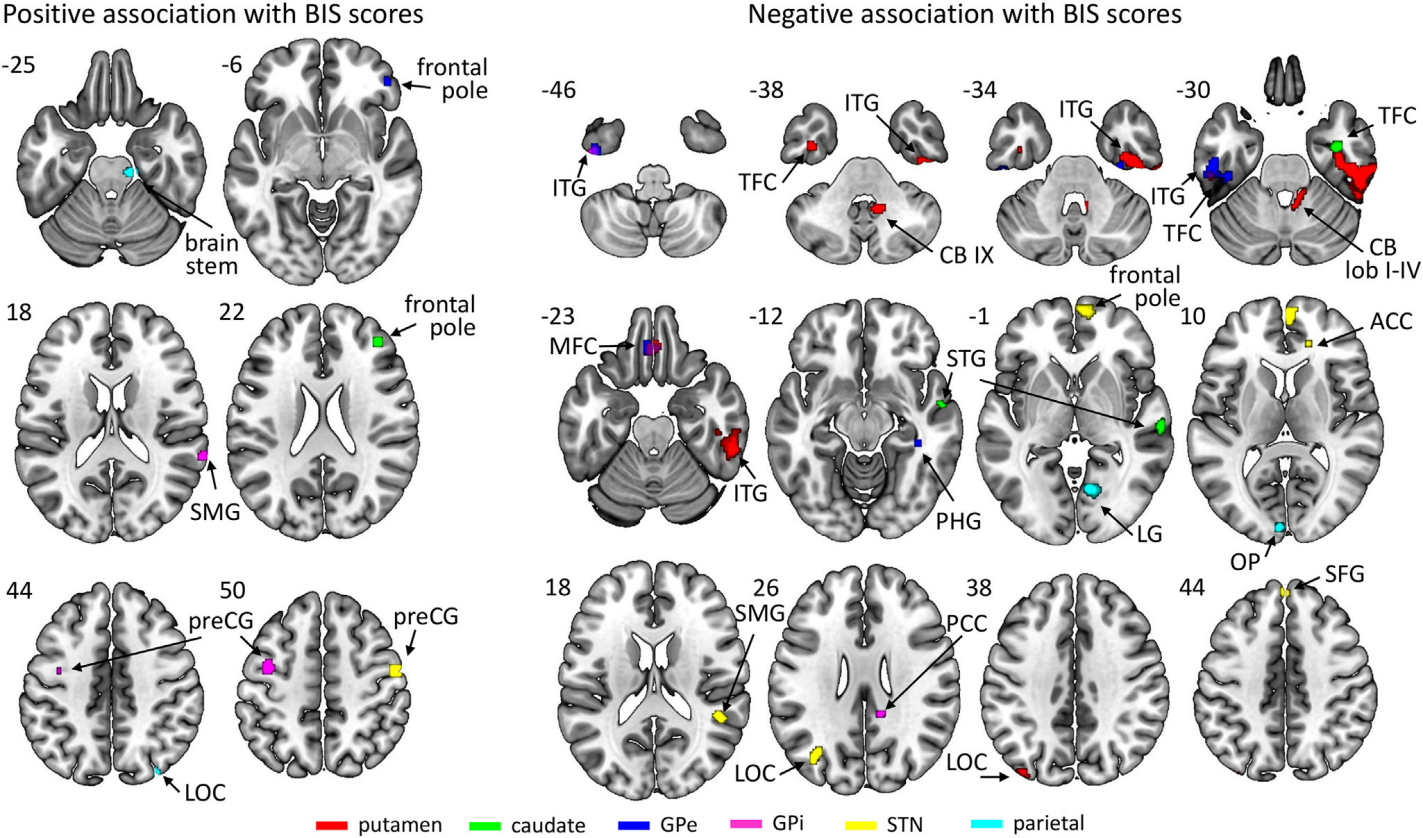
Regions of which the connectivity strength with their respective region of interest was associated positively (left) or negatively (right) with Barratt Impulsiveness Scale (BIS) scores across participants. The key for the abbreviations can be found in the notes of Table 3.

All ROIs showed significant associations between connectivity strength and BIS scores across all patients (see Figure 3; Table 3). There were a few networks in which connectivity strength was positively associated with BIS scores, but the results mainly revealed negative associations demonstrating that higher BIS scores (i.e., more impulsivity) were related to a reduced connectivity. For example, patients with higher BIS scores exhibited a weaker connectivity between the left putamen and various frontal, temporal, and cerebellar areas, between the left caudate and temporal areas, as well as between the left STN and frontal areas. We also ran separate analyses on each of the three BIS factors to evaluate associations between connectivity strength and factor scores across all patients and observed a similar pattern of results. An overview of the significant associations is presented in Tables S1–S3 in Supplementary Material.

**Table 3:** Regions of interest (ROIs) and their connected regions of which the connectivity strength was associated across all participants with Barratt Impulsiveness Scale (BIS) scores and with the number of draw choices, respectively.

	ROI	Positive association				Negative association			
		Anatomic location	Coordinates of peak	Cluster size	*Z*-score	Anatomic location	Coordinates of peak	Cluster size	*Z*-score
BIS scores	L putamen	–	–	–	–	R ITG^a^ L MFC R CB lob IX R CB lob I-IV L LOC L TFC L ITG	56, −38, −26 −2, 34, −22 12, −52, −40 10, −42, −28 −38, −84, 38 −38, −6, −38 −54, −28, −32	515 63 64 (64) 15 20 18	4.70 4.41 3.82 3.64 3.75 3.68 3.54

	L caudate	R frontal pole	40, 42, 22	21	3.62	R TFC R STG ant R STG post	36, −8, −28 56, −2, −10 62, −20, 0	27 18 19	3.90 3.64 3.54

	L GPe	R frontal pole	46, 42, −6	12	3.50	L MFC L ITG ant R PHG L ITG post L TFC R TFC	−4, 36, −24 −42, −8, −44 38, −32, −12 −54, −26, −30 −40, −28, −28 30, −24, −36	63 51 10 119 (119) 15	4.16 3.75 3.72 3.65 3.63 3.57

	L GPi	L preCG R SMG	−40, −4, 50 66, −40, 18	55 20	4.04 3.67	R PCC L ITG post	12, −40, 26 −44, −12, −48	14 27	3.98 3.70

	L STN	R preCG	52, −6, 50	42	3.83	R frontal pole R SMG L LOC R ACC L SFG	12, 62, 2 46, −42, 16 −36, −72, 28 18, 38, 12 0, 46, 44	193 45 46 11 20	4.36 4.00 3.95 3.87 3.67

	R parietal	Brain stem R LOC	14, −24, −26 34, −82, 44	31 20	4.42 3.63	R LG L OP (V1)	16, −68, −4 −4, −94, 6	147 39	4.44 3.76

Number of draw choices	L putamen	R SFG R paraCG R SFG L MTG R AG	16, 18, 58 10, 40, 20 20, 56, 16 −56, −22, −10 46, −56, 42	163 70 23 22 20	4.27 3.83 3.77 3.61 3.51	R MFC R SMG	2, 54, −32 46, −34, 52	15 13	4.10 3.72

	L caudate	L PHG R CB lob I-IV Brain stem	−18, 2, −32 12, −40, −24 −8, −38, −38	31 11 14	3.80 3.70 3.56	R PCC	18, −42, 24	12	3.98

	L GPe	R SFG R paraCG	8, 12, 58 2, 50, −6	12 12	3.60 3.48	L CALC	−28, −64, 6	26	3.86

	L GPi	R frontal pole R paraCG L SFG L ITG L MFG R ACC	16, 56, 8 12, 46, 12 −8, 32, 42 −58, −52, −24 −30, 16, 46 4, 34, 18	257 (257) 26 14 21 13	4.40 4.27 3.82 3.51 3.50 3.46	L CB lob VIII R precuneus	−14, −62, −38 14, −52, 44	33 10	4.10 3.53

	L STN	R CB lob V R CO L MCC	6, −56, −28 32, −8, 20 −10, −14, 30	11 16 18	3.74 3.60 3.54	R SFG L ITG L preCG L preCG	20, −4, 62 −44, −52, −12 −12, −14, 64 −22, −10, 62	108 17 15 10	4.38 3.62 3.51 3.37

*Cluster sizes between parentheses denote a second peak within the same cluster as listed in the row immediately preceding*.

*GPe/GPi, external/internal portion of the globus pallidus; STN, subthalamic nucleus; ITG, inferior temporal gyrus; MFC, medial frontal cortex; CB, cerebellum; LOC, lateral occipital cortex; TFC, temporal fusiform cortex; STG ant/post, superior temporal gyrus, anterior/posterior division; ITG ant/post, inferior temporal gyrus, anterior/posterior division; PHG, parahippocampal gyrus; preCG, precentral gyrus; SMG, supramarginal gyrus; PCC, posterior cingulate cortex; ACC, anterior cingulate cortex; SFG, superior frontal gyrus; LG, lingual gyrus; OP, occipital pole; V1, primary visual area; paraCG, paracingulate gyrus; MTG, middle temporal gyrus; AG, angular gyrus; CALC, calcarine cortex; MFG, middle frontal gyrus; CO, central operculum; MCC, middle cingulate cortex*.

*^a^Remained significant at FWE-corrected p < 0.05*.

**Figure 4:**
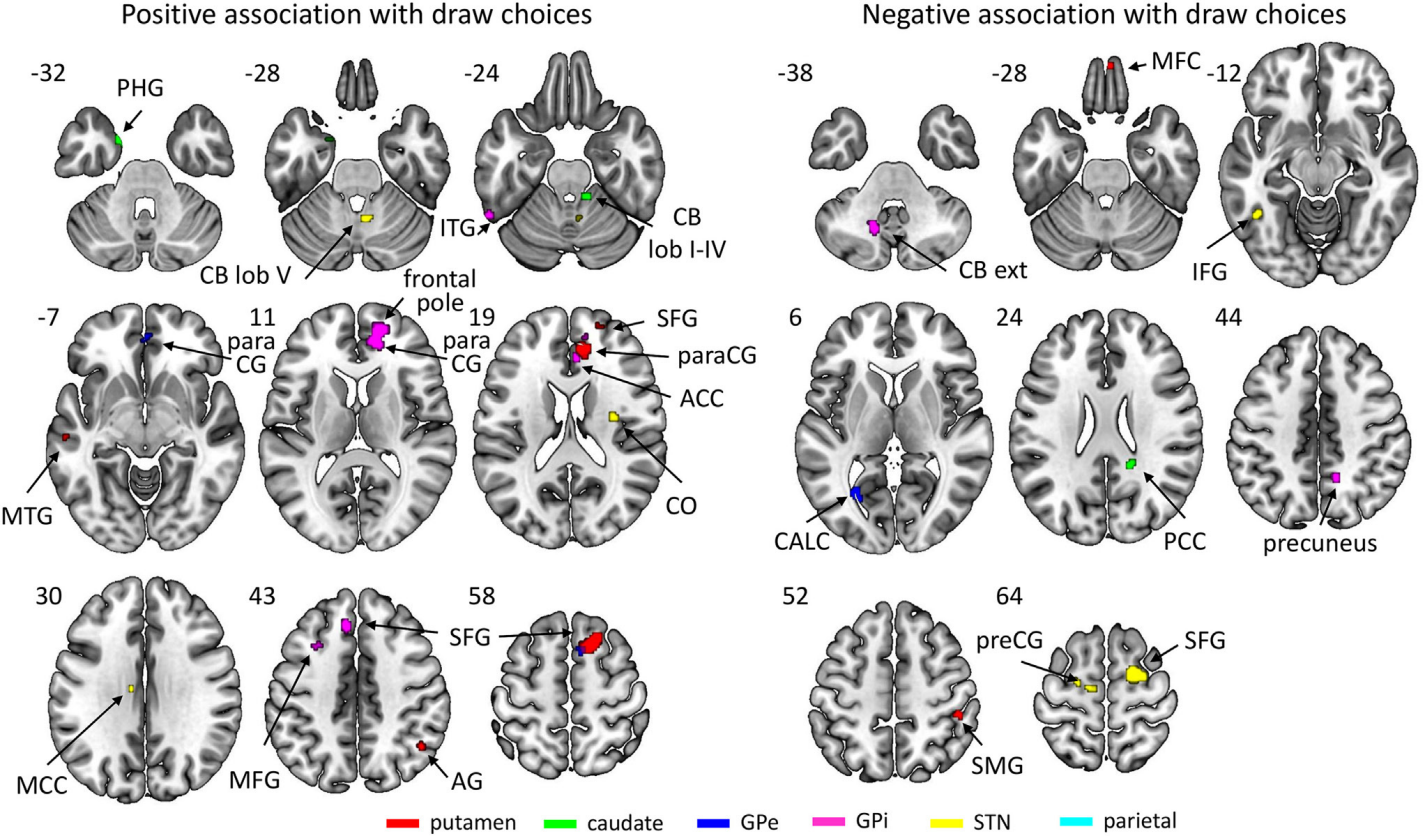
Regions of which the connectivity strength with their respective region of interest was associated positively (left) or negatively (right) with the number of draw choices across participants. The key for the abbreviations can be found in the notes of Table 3.

The basal ganglia ROIs (but not the parietal ROI; see Figure 4; Table 3) showed significant associations between resting-state network strength and behavioral performance on the beads task. The putamen, GPe, and GPi showed a stronger connectivity with frontal areas when more draw choices were made (i.e., less impulsivity).

### Structural Results

**Figure 5:**
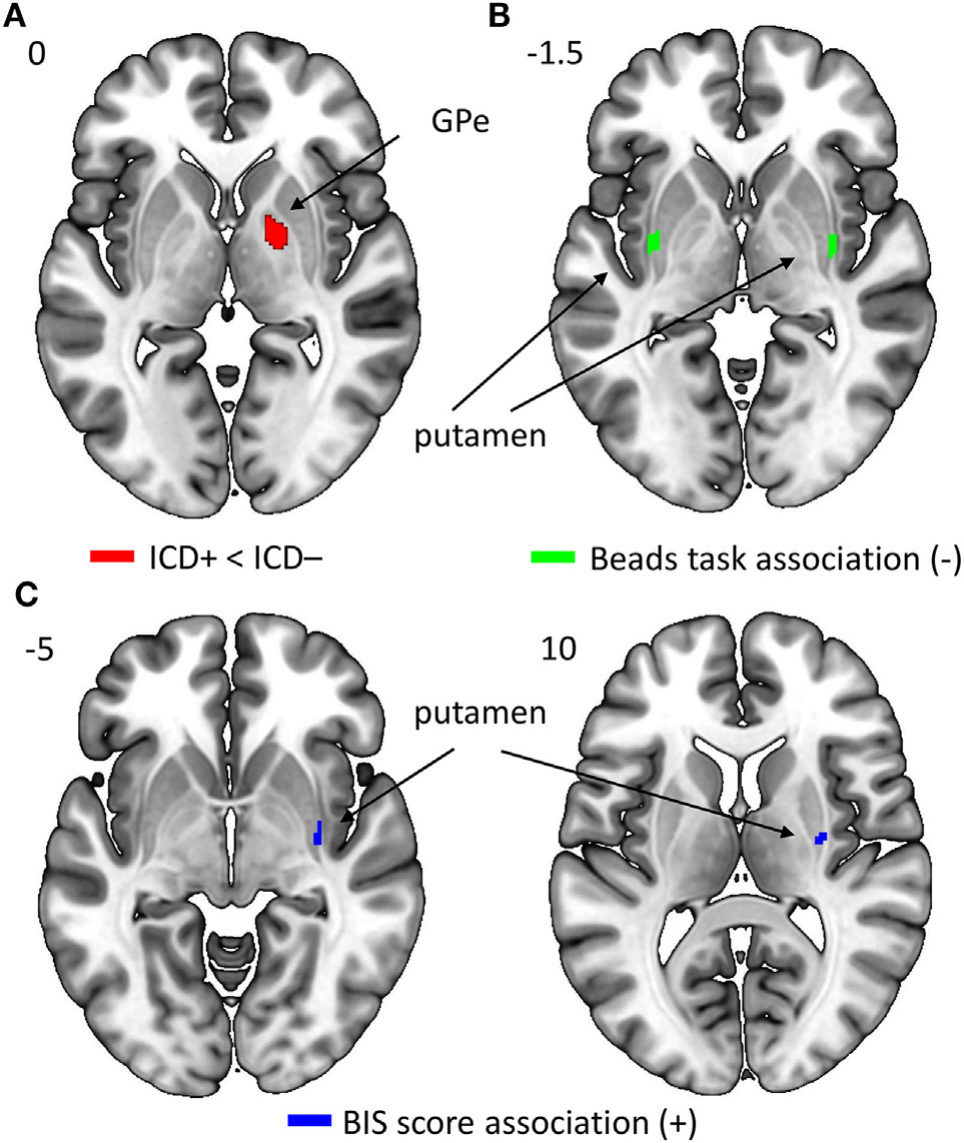
Regions in which gray matter (GM) volume was reduced in impulse control disorder (ICD)+ compared to that in ICD− patients [right GPe; panel **(A)**], in which GM volume was negatively correlated with the average number of draw choices in the beads task [bilateral putamen; panel **(B)**], or in which GM volume was positively correlated with Barratt Impulsiveness Scale (BIS) scores [right putamen; panel **(C)**].

Results revealed a significant negative association that survived FWE correction between the average number of draw choices in the beads task and GM volume in the bilateral putamen, indicating that patients who made more draw choices (i.e., less impulsivity) exhibited a smaller putamen GM volume than those who made fewer draw choices and thus collected less evidence. When evaluating results at the uncorrected threshold, we further observed that patients in the ICD+ group showed a reduced GM volume in the right GPe compared to patients in the ICD− group (see Figure 5; Table 4). There were no regions in which GM volume was significantly increased in the ICD+ group compared to that in the ICD− group. Results also showed a significantly positive association between BIS scores and GM volume in the right putamen, such that higher BIS scores (i.e., more impulsivity) were associated with a larger GM volume. We also evaluated associations between GM volume and scores on each of the three BIS factors. Only scores on the motor impulsiveness factor were associated with GM volume (see Table S4 in Supplementary Material). Specifically, results showed that higher scores on this factor were associated with a larger GM volume in the right putamen.

**Table 4:** Regions of interest (ROIs) that show differences in GM volume between the impulse control disorder (ICD)+ and ICD− groups, and ROIs showing associations between GM volume and behavioral measures [i.e., number of draw choices in the beads task and scores on the Barratt Impulsiveness Scale (BIS) questionnaire].

Contrast	Anatomic location	Coordinates of peak	Cluster size	*Z*-score
Group difference	R GPe	21, −3, 0	32	2.79

Association of GM volume and draw choices (−)	R putamen^a^ L putamen^a^	33, 2, −3 −35, −4, 0	69 46	3.79 3.73

Association of GM volume and BIS score (+)	R putamen R putamen	29, −12, 10 33, −12, −5	18 23	2.88 2.85

*Note that the association with the number of draw choices was negative, whereas the association with BIS scores was positive*.

*GM, gray matter; GPe, external portion of the globus pallidus*.

*^a^Remained significant at FWE-corrected p < 0.0.5*.

## Discussion

We examined differences in affective and sensorimotor corticostriatal functional connectivity and structural brain properties between PD patients with and without ICDs. We found that compared to patients without ICDs, the connectivity between various basal ganglia nuclei (caudate, GPe, GPi, STN) and frontal cortical areas was reduced in PD patients with ICDs. In addition, patients with ICDs showed a reduced GPe GM volume. Extending previous work, we also used behavioral assessments to examine whether individual differences in brain properties were associated with behavioral impulsivity in PD. Across all patients, we observed that *reduced* frontal–basal ganglia connectivity and *stronger* motor cortical- and cerebellar–basal ganglia connectivity were associated with more impulsivity as reflected in higher BIS scores and fewer draw choices before selecting an urn. In addition, a greater putamen volume was associated with higher BIS scores and fewer draw choices.

Our findings in combination with the literature suggest that there may be two mechanisms underlying impulsivity in PD patients. First, weaker connectivity in a frontal–striatal network may lead to impaired assessment of the reward value of actions and more risk-taking. This is in line with previous findings that ICDs in PD were associated with a reduced connectivity in cognitive and affective corticostriatal pathways (8). Dysregulation of the reward pathway may cause patients to overestimate the expected outcomes of actions and thus increase risk-taking. Second, stronger connectivity between the basal ganglia and motor areas (motor cortex and cerebellum) may result in a greater propensity to act. Besides being linked to the motor cortex, the basal ganglia are reciprocally connected with the cerebellum and involved in motor behavior [(39, 40); for a review, see Ref (41)]. Our neurostructural findings provide further support for the idea that sensorimotor basal ganglia networks contribute to impulsivity. We observed that greater GM volume of the putamen [i.e., the sensorimotor striatum; e.g., Ref. (42)] and smaller GM volume of the GPe were associated with more impulsivity. These basal ganglia regions are linked, in the sense that the putamen is known to inhibit the GPe [e.g., Ref. (43)]. As such, it is likely that greater putamen volume could be related to stronger inhibition of the GPe. The GPe in turn is part of a motor-suppressing pathway [indirect “no-go” pathway (43)], and smaller volume of this structure in PD patients with ICDs could be related to reduced inhibition of actions. Combining these notions, we speculate that greater putamen volume may more strongly inhibit the (already-smaller) GPe, resulting in less motor pathway suppression by the GPe, in turn making patients more likely to act and thus more impulsive. Together, our findings suggest that impulsivity in PD could be associated with problems in both valuation and inhibition of inappropriate behavior, although a recent review suggests that decisional rather than motor impulsivity may contribute more strongly to ICDs (44).

We further observed that ICD+ patients showed stronger striatal–cerebellar connectivity but reduced parietal–cerebellar connectivity. The cerebellum has traditionally been associated with motor functions, but cerebellar involvement in non-motor functions mediated by (among others) parietal areas has also been recognized (45). For example, cerebellar and parietal cortices are involved in response inhibition and suppression of inappropriate behavior, and changes in parietal–cerebellar connectivity are associated with poorer inhibitory control in cannabis users (46). The enhanced striatal–cerebellar connectivity we observed may subsequently cause patients to be more likely to act upon their impulses. Our findings regarding cerebellar connectivity differences between PD patients with and without ICDs fit the notion of a dual mechanism of impulsivity, with aberrant connectivity within affective parietal–cerebellar and sensorimotor striatal–cerebellar networks underlying problems in cognitive and motor control, respectively.

Our results showed no behavioral group differences in beads task performance, which contrasts with earlier findings that PD patients with ICDs drew fewer beads than patients without ICDs (16). Compared to that study, our beads task protocol was slightly modified and optimized for imaging purposes [cf Ref. (17)]. While both studies involved the same probabilities and loss amounts, subjects in the current study completed 24 trials per condition while subjects in the Djamshidian et al. (16) study completed only three trials. We repeated our behavioral analyses on just the first three trials of each condition but this did not reveal significant group differences. The demographic makeup of the patients also differed between the studies. PD patients with ICDs in Djamshidian et al. (16) study were on higher doses of medication, were younger, had an earlier age of disease onset, and a longer disease duration than the patients in the current study. In addition, the previous study showed group differences in age at PD onset, whereas the current study found no differences in clinical or demographic characteristics between groups. Importantly, patients with ICDs did show significantly higher BIS scores compared to those without ICDs, thus corroborating our classification based on the QUIP. As medication dosage affects impulsivity (6), this may explain the discrepancy in behavioral results on the beads task. Future studies should systematically examine how current age and age of disease onset might contribute to information gathering and decision making.

While two connectivity and two structural effects were significant following FWE correction, most results reported here were detected using uncorrected statistical thresholds (*p* < 0.0005 and *p* < 0.005 for the connectivity and structural analyses, respectively). As we compared two groups of PD patients, it seems reasonable that group differences are not as strong as those found when comparing patients to control subjects—especially since the two patient groups did not differ significantly in terms of current age, age of PD onset, disease duration, medication dose, and scores on the MoCA, NART-R, and UPDRS motor subscale. In addition, relative to previous studies, we used a large sample size [i.e., ≥21 patients per group, compared to ~12–20 patients per group in Ref. (8, 9, 11–13)].

A limitation of the current study is the lack of a healthy control group. However, we were interested in the effect of impulsivity on brain structural and functional connectivity changes in PD, rather than the pathophysiology of PD in general. Several prior studies have already evaluated differences in brain structure and function between PD patients and healthy control subjects. Reviews evaluating structural differences indicate that PD is typically associated with GM loss in frontal areas and basal ganglia regions (47, 48). With respect to resting-state functional connectivity, a systematic review concluded that PD patients assessed off-medication and *de novo* patients typically show a reduced corticostriatal connectivity compared to controls (49). Still, there are also indications for an increased corticostriatal connectivity in PD patients (50), as well as indications that the direction of the connectivity change may be network-specific (51). Another limitation is that all patients were tested while they were on dopamine replacement medication. Previous work has shown that medication status can modulate resting-state connectivity in PD patients [e.g., Ref. (50, 52); for a review, see Ref. (49)]. However, as medication doses did not differ between the patient groups in the present study, it is unlikely that this impacted ICD-related group differences. In addition, our approach of assessing patients on medication is in line with that of other studies investigating differences between PD patients with and without ICDs (8, 9, 11, 12, 16). Patients in the off-medication state often experience difficulties related to motor responses (and sometimes even cognitive processing), which may confound behavioral performance and thus interpretation of the task results. Finally, a technical benefit of testing patients while they were on dopamine replacement medication is that medication reduces tremor in PD patients and thus also reduces potential movement-related artifacts during scanning.

Our findings demonstrate that impulsivity in PD is associated with brain structural and functional connectivity alterations. However, they leave open the question of whether these associations reflect predispositions (i.e., neural differences existing prior to the emergence of ICDs) or whether they are related to impulsiveness-induced plasticity. Longitudinal designs may help to adjudicate these possibilities. In addition, future studies could take into account recent advances in the domain of genotyping and impulse control (53) to evaluate whether the neural characteristics observed here could potentially be associated with specific dopaminergic gene profiles that are predictive of impulsivity.

Overall, the current results corroborate that alterations in affective basal ganglia circuitries are linked to impulse control problems in PD patients [cf Ref. (8)]. Our findings show that reduced frontal–striatal connectivity and GPe volume were associated with more impulsivity. Additionally, we report the novel finding that impulsivity in PD is also linked to changes in sensorimotor striatal networks, with enhanced connectivity within this network and larger putamen volume being associated with more impulsivity. We suggest that two mechanisms may underlie impulsive behaviors in PD: one affecting decision making such that patients are more likely to select risky or inappropriate actions and one affecting sensorimotor processing such that patients are more likely to subsequently perform these actions.

## Ethics Statement

All patients provided written informed consent in accordance with the Declaration of Helsinki. The study was approved by the medical institutional review board of the University of Michigan.

## Author Contributions

BA, KC, and RS designed the study. TW organized and performed the study. MR, VK, and RS performed the data analyses. MR and RS wrote the first draft of the manuscript. All authors reviewed and edited the manuscript, and read and approved the submitted version.

## Conflict of Interest Statement

The authors declare that the research was conducted in the absence of any commercial or financial relationships that could be construed as a potential conflict of interest.
