# Supplementary material for: Impulsivity in Parkinson’s Disease Is Associated With Alterations in Affective and Sensorimotor Striatal Networks
Source: Front Neurol. 2018 Apr 26;9:279. doi: 10.3389/fneur.2018.00279 (PMC5932175; doi:10.3389/fneur.2018.00279)
Supplement: Supplementary file 1 [file table_1.docx]

Supplementary Material

Impulsivity in Parkinson’s Disease is Associated with Alterations in

Affective and Sensorimotor Striatal Networks

Marit F. L. Ruitenberg, Tina Wu, Bruno B. Averbeck, Kelvin L. Chou, Vincent Koppelmans, & Rachael D. Seidler *

*** Correspondence:** Rachael Seidler, rachaelseidler@ufl.edu

**Supplementary Table 1. Associations with attentional impulsiveness.** ROIs and their connected regions of which the connectivity strength was associated across all participants with scores on the Attentional impulsiveness factor of the BIS. Cluster sizes between parentheses denote additional peaks within the same cluster as listed in the row immediately preceding.

| ROI |  | Positive association | | | |  | Negative association | | | |
| --- | --- | --- | --- | --- | --- | --- | --- | --- | --- | --- |
|  |  | Anatomic location | Coordinates of peak | Cluster size | *Z* score |  | Anatomic location | Coordinates of peak | Cluster size | *Z* score |
| L putamen |  | - | - | - | - |  | R ITG post  R ITG post  R ITG post  L MFC  L ITG ant  L CB lob IX  L LOC  R TFC  L TTG | 56, -38, -24  46, -22, -26  46, -20, -36  -4, 36, -20  -40, -2, -42  -8, -60, -50  -40, -84, 36  28, -32, -26  -44, -18, 2 | 325  (325)  (325)  52  69  34  28  17  18 | 4.59  3.69  3.65  4.16  4.12  4.05  3.91  3.68  3.62 |
| L caudate |  | R AG  L SFG | 34, -52, 28  -14, 0, 72 | 24  21 | 3.93  3.59 |  | R OFC  R STG ant  R temporal pole  L AG  R STG post  L OFC  Commissure post | 36, 26, -16  56, 2, -10  22, 10, -28  -48, -54, 14  64, -18, -2  -36, 28, -18  0, 10, -32 | 44  30  27  57  44  11  11 | 4.26  3.98  3.85  3.81  3.62  3.51  3.46 |
| L GPe |  | - | - | - | - |  | L ITG ant  L SFG  L CB lob VIIIa  L TFC  L ITG post  L ITG post  R frontal pole  L LOC  R MFC | -44, -6, -44  -26, 34, 52  -4, -62, -32  -40, -28, -28  -54, -26, -34  -52, -16, -30  24, 36, 48  -38, -84, 38  2, 40, -18 | 121  23  18  27  108  (108)  20  11  18 | 4.29  3.84  3.79  3.77  3.76  3.52  3.71  3.63  3.55 |
| L GPi |  | L LOC  L preCG  L frontal pole | -32, -80, 22  -40, -6, 50  -6, 50, -32 | 59  90  15 | 4.10  4.07  3.42 |  | R postCG  L frontal pole  L precuneus  L ITG post  R paraCG | 10, -40, 26  -4, 66, 0  -8, -54, 20  -54, -18, -30  2, 38, -10 | 50  21  36  27  39 | 4.56  3.63  3.62  3.58  3.54 |
| L STN |  | R preCG  L SPL  L SPL | 50, -10, 50  -24, -44, 48  -34, -42, 70 | 38  21  11 | 3.94  3.66  3.52 |  | R frontal pole  R SFG  R SFG  R frontal pole  R frontal pole  L LOC | 12, 44, 44  2, 44, 46  20, 12, 70  12, 62, 2  4, 64, 6  -36, -70, 28 | 289  (289)  32  74  (74)  11 | 4.83  4.54  4.09  3.97  3.55  3.66 |
| R parietal |  | - | - | - | - |  | R LG  L occipital pole  R ACC | 12, -66, -6  -4, -94, 4  2, -14, 26 | 111  20  10 | 4.06  3.75  3.45 |
| GPe/GPi = external/ internal portion of the globus pallidus; STN = subthalamic nucleus; ITG ant/post = inferior temporal gyrus, anterior / posterior division; MFC = medial frontal cortex; CB = cerebellum; LOC = lateral occipital cortex; TFC = temporal fusiform cortex; TTG = transverse temporal gyrus; AG = angular gyrus; SFG = superior frontal gyrus; OFC = orbitofrontal cortex; STG ant/post = superior temporal gyrus, anterior / posterior division; preCG = precentral gyrus; postCG = postcentral gyrus; ; paraCG = paracingulate gyrus; SPL = superior parietal lobule; LG = lingual gyrus; ACC = anterior cingulate cortex | | | | | | | | | | |

**Supplementary Table 2. Associations with motor impulsiveness.** ROIs and their connected regions of which the connectivity strength was associated across all participants with scores on the Motor impulsiveness factor of the BIS. Cluster sizes between parentheses denote additional peaks within the same cluster as listed in the row immediately preceding.

| ROI |  | Positive association | | | |  | Negative association | | | |
| --- | --- | --- | --- | --- | --- | --- | --- | --- | --- | --- |
|  |  | Anatomic location | Coordinates of peak | Cluster size | *Z* score |  | Anatomic location | Coordinates of peak | Cluster size | *Z* score |
| L putamen |  | L MTG | -58, -58, -8 | 18 | 3.63 |  | R CB lob X  R ITG post  L TFC ant  L TFC post  L MFC  R OFC  R TFC post  R TFC post  R ITG post  R ITG post | 8, -48, -30  56, -38, -24  -38, -8, -38  -36, -12, -46  -2, 34, -22  14, 18, -30  38, -18, -44  42, -16, -36  44, -22, -28  60, -24, -30 | 83  99  61  (61)  25  24  114  (114)  (114)  32 | 4.35  4.24  3.99  3.44  3.86  3.86  3.78  3.75  3.56  3.67 |
| L caudate |  | L frontal pole | -18, 44, 16 | 12 | 3.78 |  | R STG ant  R STG ant  L MTG  R CB crus II | 56, -2, -8  64, -8, -6  -44, -52, 2  44, -76, -48 | 69  (69)  11  13 | 4.33  3.55  3.59  3.51 |
| L GPe |  | R frontal pole  R frontal pole  R IFG  R frontal pole | 48, 42, -6  44, 38, 4  54, 26, 2  34, 46, 22 | 44  (44)  22  17 | 3.76  3.59  3.56  3.42 |  | L MFC  R OFC  L OFC  L TFC post  L OFC  R ITG post | -4, 34, -24  16, 18, -26  -26, 26, -22  -38, -14, -46  -20, 34, -24  64, -22, -26 | 126  137  19  62  21  25 | 4.54  4.08  3.93  3.92  3.69  3.55 |
| L GPi |  | - | - | - | - |  | R SMG  L CB crus I | 38, -32, 36  -40, -74, -22 | 32  17 | 3.96  3.64 |
| L STN |  | L PT  R preCG  Brain stem | -40, -30, 12  54, -2, 52  -8, -32, -46 | 13  12  11 | 3.64  3.60  3.57 |  | R SMG  R MFG  R LOC  R PO  R CB lob VI  L SFG  L CB crus I  L LOC | 46, -42, 16  30, 4, 44  46, -60, 14  50, -30, 30  22, -46, -42  -2, 48, 44  -38, -68, -24  -36, -70, 24 | 57  37  71  60  13  27  11  10 | 4.08  4.03  4.01  3.88  3.84  3.71  3.60  3.48 |
| R parietal |  | R LOC  R OFC | 34, -82, 44  26, 18, -26 | 61  24 | 4.44  3.92 |  | L postCG  R LG | -64, -8, 12  16, -68, -4 | 24  14 | 3.82  3.55 |
| GPe/GPi = external/ internal portion of the globus pallidus; STN = subthalamic nucleus; MTG = middle temporal gyrus; CB = cerebellum; ITG ant/post = inferior temporal gyrus, anterior / posterior division; TFC = temporal fusiform cortex; MFC = medial frontal cortex; OFC = orbitofrontal cortex; STG ant/post = superior temporal gyrus, anterior / posterior division; IFG = inferior frontal gyrus; SMG = supramarginal gyrus; PT = planum temporale; preCG = precentral gyrus; MFG = middle frontal gyrus; LOC = lateral occipital cortex; PO = parietal operculum; SFG = superior frontal gyrus; postCG = postcentral gyrus; LG = lingual gyrus | | | | | | | | | | |

**Supplementary Table 3. Associations with non-planning impulsiveness.** ROIs and their connected regions of which the connectivity strength was associated across all participants with scores on the Non-planning impulsiveness factor of the BIS. Cluster sizes between parentheses denote additional peaks within the same cluster as listed in the row immediately preceding.

| ROI |  | Positive association | | | |  | Negative association | | | |
| --- | --- | --- | --- | --- | --- | --- | --- | --- | --- | --- |
|  |  | Anatomic location | Coordinates of peak | Cluster size | *Z* score |  | Anatomic location | Coordinates of peak | Cluster size | *Z* score |
| L putamen |  | L CB crus I | -48, -64, -32 | 18 | 3.60 |  | L CB lob VI  R ITG  R ITG post  L MFC  L MTG  L ITG post  L LOC | 30, -24, -38  58, -40, -28  52, -30, -30  -2, 34, -22  -46, -2, -26  -56, -28, -30  -36, -84, 40 | 104  108  (108)  32  25  37  13 | 4.18  3.96  3.78  3.93  3.80  3.78  3.73 |
| L caudate |  | R frontal pole  R PCC | 36, 40, 20  8, -36, 8 | 60  18 | 4.08  3.63 |  | R OFC  R TFC  R TFC  L LOC | 14, 18, -28  36, -8, -28  32, -24, -34  -38, -82, 40 | 36  20  19  11 | 3.85  3.78  3.69  3.47 |
| L GPe |  | R temporal pole  R LOC | 30, 14, -30  48, -84, 10 | 13  12 | 3.53  3.47 |  | R TFC  L MTG  R CB crus I | 30, -24, -36  -48, -60, 12  46, -62, -20 | 66  10  12 | 4.36  3.66  3.60 |
| L GPi |  | R caudate  L preCG  R frontal pole  R SMG  R amygdala | 18, 2, 18  -40, -2, 52  16, 48, -10  68, -40, 18  22, -8, -12 | 17  32  12  18  15 | 3.89  3.81  3.73  3.72  3.61 |  | - | - | - | - |
| L STN |  | R caudate | 10, -10, 26 | 13 | 3.69 |  | R frontal pole  R frontal pole  R ACC  R LOC  R SMG  L LOC | 10, 62, 2  2, 58, 10  18, 38, 10  36, -66, 20  46, -42, 16  -32, -68, 26 | 188  (188)  (188)  42  14  22 | 4.12  3.67  3.58  3.93  3.65  3.59 |
| R parietal |  | Brain stem | 14, -26, -26 | 36 | 4.46 |  | R LG  R CB lob VI  R CB lob VI | 16, -68, -4  24, -72, -18  16, -74, -18 | 206  (206)  (206) | 3.92  3.85  3.81 |
| GPe/GPi = external/ internal portion of the globus pallidus; STN = subthalamic nucleus; CB = cerebellum; ITG ant/post = inferior temporal gyrus, anterior / posterior division; MFC = medial frontal cortex; MTG = middle temporal gyrus; LOC = lateral occipital cortex; PCC = posterior cingulate cortex; OFC = orbitofrontal cortex; TFC = temporal fusiform cortex; preCG = precentral gyrus; SMG supramarginal gyrus; ACC = anterior cingulate cortex; LG = lingual gyrus | | | | | | | | | | |

**Supplementary Table 4.** ROIs that show associations across all participants between gray matter (GM) volume and scores on the three BIS factors. Cluster sizes between parentheses denote additional peaks within the same cluster as listed in the row immediately preceding.

| Association GM volume with | Anatomic location |  | Positive association | | |
| --- | --- | --- | --- | --- | --- |
|  |  |  | Coordinates of peak | Cluster size | *Z* score |
| Motor impulsiveness | R putamen  R putamen  R putamen |  | 30, 2, 12  29, 11, 10  29, -10, 10 | 74  (74)  (74) | 3.16  3.06  3.04 |
